# Factors Affecting the Choice of a Career in the Field of Surgery Among Medical Students of Karachi

**DOI:** 10.7759/cureus.3542

**Published:** 2018-11-04

**Authors:** Farhan Zaheer, Hafeez Ur Rehman, Wajahat Fareed, Mohammad O Khan, Syed Asad Hasan Rizvi

**Affiliations:** 1 Surgery, Civil Hospital, Dow University of Health Sciences, Karachi, PAK; 2 Surgical, Civil Hospital, Dow University of Health Sciences, Karachi, PAK; 3 Internal Medicine, Civil Hospital, Dow University of Health Sciences, Karachi, PAK

**Keywords:** surgery, career choice, medical students, karachi, interest, factors, academic

## Abstract

Introduction

Medical students choose to pursue their careers based on multiple internal and external factors. These factors, in turn, not only affect their personal future but the overall status of the health care system of their country. A recent decline in the interest of medical students towards the surgical career is being observed and, therefore, the factors influencing their choice need to be evaluated. We aimed to identify these factors in medical students of a public sector university of Karachi.

Methods

A cross-sectional study was conducted at Dow Medical College from April 2018 to May 2018. A pre-tested questionnaire was administered to a sample of 250 students. Besides sociodemographic factors, the choice of career was identified and the factors that influenced it were assessed. Students' opinions were collected using a Likert scale. Data were entered and analyzed using the IBM Statistical Package for the Social Sciences 23.0 (IBM, NY, USA). Frequencies were calculated for individual variables. The chi-square test was used to measure statistical differences between categorical variables and a p-value of <0.05 was considered to be significant.

Results

In this study, 224 out of 250 questionnaires were returned with complete data, yielding a response rate of 89.6%. We found that 48.2% of students reported a desire to pursue a career in surgery. Students whose fathers were more qualified and belonged to the field of health care were more likely to pick a surgical career (p-value of 0.034 and 0.039, respectively). Students who were willing to pursue a path in surgery more often thought that the social standing of surgeons had its importance (p=0.037). These students also agreed that high salary has a role to play in affecting the choice of career (p=0.023). The most common factors that encouraged students for the choice of a surgical career included the practical implication of skills (57.4%) followed by an academic interest in the field (53.7%), and high income (42.6%). The most common discouraging factors included lifestyle and long working hours (56.9%), followed by less academic interest (31%).

Conclusion

Our study provides a valuable insight into the factors that influence the choice of medical students for pursuing a career in surgery. We also identified the factors that demotivated them from doing so. In our opinion, better incorporation of surgery into the curriculum, proper attention given to students during their surgical rotation and restructuring of the surgical training program are some of the ways that may improve the interest of students in the field of surgery.

## Introduction

Throughout the past decade, there has been a rising concern about a decline in the number of medical students who wish to pursue a career in the field of Surgery [1]. This is indicated by several studies conducted worldwide, most notably from South Africa [2], Australia [3], Ireland [4], and the USA [5]. Multiple factors have been reported to contribute to this trend. In the present literature, these factors that influence students to pick a path in the surgical career have been broadly classified into internal factors, such as gender and academic interest, and external factors, such as mentors, salary, and competitive training programs [6-8]. Given that the opinions and experiences shared during undergraduate training influences the students with their future career choice [9], and that these very choices not only affect their personal future but the overall status of the health care system of their country [10], it is essential to identify the factors that shape the students’ choices and to use the data to bring forth improvements to the system where possible.

Although a handful of studies have been conducted in Pakistan [11, 12], the overall data on this issue still remains scarce. This study aimed to assess the popularity of the field of surgery among the students of a public sector university in Karachi and to identify the factors that affect their decision.

## Materials and methods

A cross-sectional study was conducted at Dow Medical College from April 2018 to May 2018 after obtaining approval from the institutional review board of Dow University of Health Sciences. A pre-tested questionnaire that consisted of four parts was used. The first part included questions regarding the sociodemographic characteristics of the participants, including age, gender, marital status, year of study, parents' qualification, and parents' profession. The second part consisted of seven Likert items that included statements that were used to assess the participant’s opinions on the factors that encouraged or discouraged students to pursue a career in surgery. These factors included social standing of surgeons, high salary, low mortality rate, longer duration of surgical training, longer working hours, male dominance in the field, and lack of competent surgical training programs in the country. Its third part was designed to be filled by students who wanted to pursue surgery in the future and involved the assessment of the factors which motivated them to do so. The final part was designed to be filled by students who did not want to pursue surgery and involved assessment of the factors which affected their decision.

All students enrolled in the five-year Bachelor of Medicine, Bachelor of Surgery (MBBS) program at Dow medical college were included in the study. Students, who had graduated or had been enrolled in any program other than MBBS, were excluded. A pilot study was performed on 20 participants in order to reduce bias, minimize flaws, and improve understanding. Furthermore, no imputation of missing data was carried out.

A convenience sampling method was employed to distribute 250 questionnaires with informed consent. Of these 224 were returned with complete data, yielding a response rate of 89.6%. Data were entered and analyzed using the IBM Statistical Package for the Social Sciences 23.0 (IBM, NY, USA). Frequencies were calculated for individual variables and were represented as percentages in tables and figures. The chi-square test was used to measure statistical differences between categorical variables and a p-value of <0.05 was considered to be significant.

## Results

Table 1 summarizes the demographic characteristics of the participants. Most of the participants were aged 20 years or above with a male to female ratio of almost 1:3. Around three-quarters of the students belonged to either the first or the third year of study. An overwhelming majority of the participants had fathers who were graduates or higher; however, just slightly more than two-fifths of them belonged to the field of health care. When it came to the mother’s qualification, almost three-quarters were graduates or higher and slightly less than two-fifths belonged to the field of health care. When comparing these demographic factors with the likelihood of the participant wishing to pursue a future career in surgery, it was found that students whose fathers were more qualified and belonged to the field of health care were more likely to pick a surgical career (p-value of 0.034 and 0.039, respectively). No other correlation between demographic variables and pursuing surgery was found.

**Table 1:** Sociodemographic characteristics of participants *Doctor of Philosophy

Sociodemographic variables	Classes	Frequencies	p-value
Age	Below 20	82 (36.6%)	>0.05
	Above 20	142 (63.4%)	
Gender	Male	73 (32.6%)	>0.05
	Female	151 (67.4%)	
Year of Study	First	58 (25.9%)	>0.05
	Second	6 (2.7%)	
	Third	124 (55.4%)	
	Fourth	30 (13.4%)	
	Fifth	6 (2.7%)	
Father's Qualification	Matric	4 (1.8%)	0.034
	Inter	8 (3.6%)	
	Graduation	142 (63.4%)	
	Masters	66 (29.5%)	
	PhD*	4 (1.8%)	
Father's Profession	Medical	49 (21.9%)	0.039
	Non-Medical	175 (78.1%)	
Mother's Qualification	Matric	14 (6.3%)	>0.05
	Inter	38 (17.0%)	
	Graduation	130 (58.0%)	
	Masters	40 (17.9%)	
	PhD*	2 (0.9%)	
Mother's Profession	Medical	38 (17%)	>0.05
	Non-Medical	186 (83.0%)	

Table 2 summarizes the responses to the Likert items on the opinions of students about the factors that influence people to pick a surgical career. Almost a quarter of the students agreed that the social standing that surgeons enjoy had its significance. Students who were willing to pursue a path in surgery were more likely to agree with this statement than the rest (p=0.037). Majority of the students were seen to either agree or strongly agree that high salary was a major influencing factor that affects the decision of pursuing surgery as a career. Students who wanted to pursue surgery in the future were more likely to agree to this notion as compared to the ones who did not (p=0.023). Almost half of the students remained neutral to the idea that better surgical outcomes and lesser mortality rates in the surgical field contribute to affect the decision; however in the remaining half, more than one-quarter of the students did agree to this.

**Table 2:** Opinion of students regarding factors that influence the choice of future surgical career *p-value indicates the relationship between the factors and the choice of surgical career

Statements	Strongly Disagree	Disagree	Neutral	Agree	Strongly Agree	*p-value
The social standing and status that the surgeons enjoy drives the students' interest towards a career in surgery.	20 (8.9%)	10 (4.5%)	104 (46.4%)	72 (32.1%)	18 (8.1%)	0.037
High salary is an influencing factor in pursuing surgery as a career.	14 (6.3%)	20 (8.9%)	55 (24.6%)	97 (43.3%)	38 (17.0%)	0.023
Immediate improvement in patient's health after surgical intervention/low mortality rate can be an influencing factor.	4 (1.8%)	26 (11.6%)	110 (49.1%)	66 (29.5%)	18 (8.0%)	0.9
The comparatively longer duration of training makes students apprehensive about choosing surgery as a discipline.	8 (3.6%)	34 (15.2%)	78 (34.8%)	88 (39.3%)	16 (7.1%)	0.7
Long working hours influence a student's decision to pursue surgery.	10 (4.5%)	42 (18.8%)	52 (23.2%)	102 (45.5%)	18 (8.0%)	0.358
The male dominance in the field of surgery discourages women from pursuing it.	22 (9.8%)	70 (31.3%)	74 (33.0%)	40 (17.9%)	18 (8.0%)	0.219
There is a lack of competent surgical training programs in our country.	6 (2.7%)	22 (9.8%)	72 (32.1%)	82 (36.6%)	42 (18.8%)	0.717

In the questions assessing discouraging factors towards a surgical career choice, around half of the students agreed that the longer duration of the surgical training course played a major role. Slightly more than half agreed that the long working hours associated with the field of surgery was a discouraging factor. Majority of the students were seen to either disagree or remain neutral to the notion that male dominance in the field of surgery discouraged women in pursuing it as a career, whereas only a quarter agreed to this. Even though around one-third of the study participants remained neutral to the statement that competent surgical training programs are present in their country, only one-tenth of the students were seen to disagree with this point of view.

We found that 48.2% of the students reported a desire to pursue the field of surgery as a career. Table 3 summarizes the factors that are claimed to be encouraging by these students who aspire to be future surgeons. The most common factor amongst these students was the practical implication of skills (57.4%), followed by an academic interest in the field (53.7%). A vast majority of these students claimed that accessible role models and immediate improvement in the patients’ health after surgical intervention were not among the core reasons that influenced their decision. Around two-thirds were seen to disagree that the lifestyle or the social standing that the surgeons enjoy, were the factors that have an effect on their decision.

**Table 3:** Factors that encourage the choice of pursuing future surgical career

Factors	Yes	No
Academic interest	58 (53.7%)	50 (46.3%)
Salary	46 (42.6%)	62 (57.4%)
Lifestyle	42 (38.9%)	66 (61.1%)
Accessible role models	16 (14.8%)	92 (85.2%)
Immediate improvement in patient's health after surgical interventions/low mortality rate	20 (18.5%)	88 (81.5%)
Social position/standing	38 (35.2%)	70 (64.8%)
Practical application of skills	62 (57.4%)	46 (42.6%)

Table 4 summarizes the factors that were claimed by medical students to discourage them. The most commonly identified reason was that of the busy lifestyle and long working hours, as claimed by more than half of the students, followed by less academic interest. Females were less likely to claim as compared to males that less academic interest in the field of surgery influenced them not to pursue a surgical career (p = 0.04). Slightly less than a quarter claimed that it was due to the long duration of the surgical training course; however, females were seen to present this reason more than males (p=0.013). Only a negligible number of students said that male dominance, lack of a competent surgical program, salary, and lack of role models were contributing factors. However, the lack of a competent surgical program seemed to be a reason expressed more by females (p=0.022).

**Table 4:** Factors that discourage the choice of pursuing future surgical career

Factors	Yes	No
Duration of training	17 (29.3%)	41 (70.7%)
Lack of competent surgical training program	8 (13.8%)	50 (86.2%)
Lack of role models/mentors	4 (6.9%)	54 (93.1%)
Lifestyle/working hours	33 (56.9%)	25 (43.1%)
Male dominant field	2 (3.4%)	56 (96.6%)
No academic interest	18 (31.0%)	40 (69.0%)
Salary/monetary returns	5 (8.6%)	53 (91.4%)

## Discussion

Our study was conducted specifically to quantify information related to the field of surgery, its popularity of being chosen as a future career by medical students, and the factors involved in shaping one's opinion related to it.

In our study, 48.2% of the students reported a desire to pursue surgery as a career. This finding falls just slightly short of the findings of Rehman et al. [11], where the percentage was noted to be 50.3% in a pool of medical students of Karachi coming from both public and private institutes. Our results were in contrast to those reported by Huda et al. [13], which reported it to be only 21% in a group of final year medical students enrolled in a private institution in Karachi. Another study involving fourth-year medical students in Lahore showed that only 33% of the students were interested in a surgical career [12]. The wide variation in interest across these studies may be attributed to the amount of exposure that different institutes offer in the field of surgery and the amount of attention the students are being given in their surgical rotations. The competence level and facilities of the surgical departments across institutes might be different and some might have better role models available than others. The year of study and the institute being private or public might have also played a role in the variation present as also noted by other authors [14].

Our results showed that students whose fathers were more qualified and belonged to the field of health care were more likely to pick a surgical career. These findings contradict the international observations of a similar study involving students at the University of Cape Town (UCT), where no such correlation with interest in surgery and parental qualification or profession was found [15]. Several studies, unspecific to the field of surgery, do highlight parental influence as one of the major factors involved in shaping career choices in students [11, 16]. The availability of a role model in the immediate family could be one of the reasons for these results. These students would have had an exposure to the field since childhood while visiting the surgical facilities with their parents. Proper career guidance from parents and a clear set-out path, would have all contributed to the reason why these students were found to be more interested in the field of surgery.

In our study, it was a highly popular opinion that a prestigious social standing and high salary were the main reasons that attract the students towards the field of surgery. These factors were identified as being influential, more often by the students who desire to pursue a surgical career as compared to those who did not. Multiple other studies have highlighted the significance of these two factors and it is not surprising to observe the same trend in Karachi as well [17,18]. When the students who desired to pursue the surgical career were inquired about the reasons for their decision, the three most frequently identified reasons were academic interest, practical application of skill, and a high income. These results are in line with other studies that have also shown similar trends, in which personal interest was the most influential factor [13].

The students who did not want to pursue surgery identified long working hours, no academic interest, and longer duration of training as the most frequent reasons for shaping their choice. These findings are in line with a similar international study in which lifestyle and working hours were the two most common reasons that discourage students from pursuing a career in surgery [15]. It is important to note, however, that females were less likely to present lack of academic interest as a reason, although significantly more of them highlighted the long duration of training as a major concern. Even though other studies show that there is a correlation between gender and choosing surgery as a career [4,19,20], our study found no such evidence for this to be true. This might be due to the increasing awareness campaigns being conducted for women empowerment and women education, which have motivated females to become more career-oriented rather than getting engaged in domestic responsibilities. Our study does, however, highlight that one of the major barriers which discourage females to pursue a career in surgery is the long duration of training. Again, the reason behind this could reside in pressure to engage with domestic responsibilities than to focus on careers.

Although done with immense credibility, there are some limitations to our study. Firstly, a convenience sampling method was used, giving a disproportionate cluster of students from each year. Secondly, our study was only conducted on the students of Dow Medical College and did not take into consideration students from other medical colleges. Further multi-centered studies that use cluster sampling might bring forth a better snapshot of the status quo.

## Conclusions

In conclusion, the field of surgery has maintained its interest throughout the student body with only a slight decrease seen from previous surveys in Karachi. It also seems to be a more popular choice in government universities as compared to other institutes in Karachi or other cities. The most important motivational factors seem almost similar to those found in other studies, such as high income and academic interest while the demotivating factors revolve around a longer duration in training and no academic interest. In our opinion, better incorporation of surgery into the curriculum, proper attention given to students during their surgical rotation, and renovation of the surgical training program are some of the ways that may improve the interest of students in the field of surgery.
